# Oak seedling microbiome assembly under climate warming and drought

**DOI:** 10.1186/s40793-024-00602-4

**Published:** 2024-08-28

**Authors:** Daniel Hoefle, Milena Sommer, Birgit Wassermann, Maria Faticov, Demetrio Serra, Gabriele Berg, Ayco J.M. Tack, Ahmed Abdelfattah

**Affiliations:** 1 Leibniz Institute for Agricultural Engineering and Bioeconomy (ATB), Max-Eyth Allee 100, 14469 Potsdam, Germany; 2 Institute of Environmental Biotechnology, Graz University of Technology, Petersgasse 12, Graz, 8010 Austria; 3 Bolin Centre for Climate Research, Stockholm University, Svante Arrhenius väg 20A, Stockholm, SE-106 91 Sweden; 4 Département de biologie, Université de Sherbrooke, Sherbrooke, QC J1K 2R1 Canada; 5 Fondazione Mediterranea Terina Onlus, Zona industriale Benedetto XVI, 88046 Ficarella, CZ Italy; 6 Institute for Biochemistry and Biology, University of Potsdam, 14476 Potsdam OT, Golm Germany; 7 Department of Ecology, Environment and Plant Sciences, Stockholm University, Svante Arrhenius väg 20A, Stockholm, SE-106 91 Sweden

**Keywords:** Climate change, Microbiome assembly, *Quercus robur L.*, Phyllosphere, Rhizosphere

## Abstract

Despite that climate change is currently one of the most pervasive challenges, its effects on the plant-associated microbiome is still poorly studied. The aim of this study was to evaluate the impact of the independent and combinatory effect of climate warming and drought on the microbiome assembly of oak from seed to seedling. In a multifactorial experimental set up, acorns were subjected to different temperatures (15 °C, 20 °C, and 25 °C) and soil moisture levels (drought (15%) and control (60%)) from germination until the seedling stage, after which the bacterial and fungal communities associated to the rhizosphere and phyllosphere were characterized by amplicon sequencing and qPCR. The results showed a stronger effect of temperature on fungal than on bacterial diversity and the effect was more pronounced in the phyllosphere. Under drought condition, temperature had a significantly negative effect on phyllosphere fungal diversity. In the rhizosphere, temperature had a significant effect on the fungal community composition which was primarily caused by species turnover. Regardless of temperature, *Actinobacteriota* was significantly enriched in drought, a group of bacteria known to increase plant drought tolerance. This study provides new insights into the effect of climate change on the plant microbiome in natural ecosystems.

**Supplementary Information:**

The online version contains supplementary material available at 10.1186/s40793-024-00602-4.

## Introduction

Climate change has led to increasing occurrences of extreme weather events such as heat waves and drought, changes in precipitation patterns, and disruptions of many other environmental patterns [34]. Those changes result in biodiversity loss and alter species’ life cycle events and their spatial distribution in general, affecting ecosystem multifunctionality and stability [4, 12, 67]. Most studies on the effect of climate change so far have focused on macro-organisms such as plants and animals [30, 54]. However, climate change is also expected to affect the richness, diversity and community composition of microbial communities associated with plants [6, 43, 50]. Additionally, the majority of studies about the effects of climate change on the plant microbiome have focused on annual crops [57, 70, 75], whereas less studies have focused on trees, despite their invaluable importance for global ecosystem functioning [7, 25, 31]. While we have a good understanding of the effect of warming and drought on the plant physiology and fitness, we lack insights into the independent and the combinatory effects of warming and drought on the plant-associated microbiome.

The effects of rising temperatures on the plant microbiome vary depending on the studied organisms (e.g., bacteria or fungi) and plant compartments (e.g., rhizosphere or phyllosphere) [5, 7, 31, 70]. For instance, warming was shown to increase bacterial richness and diversity in the phyllosphere of herbaceous annual plants and grapevines [5, 17]. Further, the bacterial community composition changed in the rhizosphere due to warming [74]. However, members of the plant microbiome seem to respond differently to changing temperatures. For example, warming reduced the abundance of beneficial bacteria such as *Sphingomonas* spp. and *Rhizobium* spp., and increased the abundance of *Buchnera* and *Wolbachia* spp. in the phyllosphere of *Galium album* [5] and of members of *Actinobacteria* in the rhizosphere of *Sorghum* [70]. Phyllosphere fungal species richness was decreased due to warming in oak [31], poplar [7] and olive trees [28]. In the rhizosphere, warming increased fungal diversity and abundance on rice [74], while the fungal composition, richness or evenness on arctic willow was not affected [32].

Besides warming, drought is an important abiotic factor affecting plants and their microbiome [21, 34]. Because of their different physiology, bacterial and fungal communities are expected to respond differently to drought [8, 24, 52]. Bacterial diversity and abundance were reported to increase under drought conditions in soybean [73], while the leaf bacterial richness was reduced in grasses and tomato [11, 24]. The plant rhizosphere microbiome shifts in favour of *Actinobacteria* and other Gram-positive taxa, which compete against many Gram-negative taxa that are typically found in the rhizosphere [27, 47, 59, 71]. Regarding the phyllosphere, on the other hand, the abundance of *Gammaproteobacteria* was reported to increase under severe drought [11]. With regard to fungi, root-associated fungal diversity [3], as well as phyllosphere fungal diversity [24] were reported to increase in drought conditions. The composition of both bacterial and fungal root-associated communities shifted under drought, with the effect being stronger for bacteria than fungi [14], while in phyllosphere drought only affected the bacterial community composition of oak [51]. In summary, warming and drought can have varying impacts on the rhizosphere and phyllosphere microbiota of different plant species, which we summarized in Table 1. Here, only one study investigated the combinatory effect of warming and drought [70], which is, however, an important aspect since warming can enhance the effect of drought and vice versa [34, 51, 61], accelerating the impact on microbial composition and diversity. In this context, the aim of this study was to address the following question: *What is the effect of temperature*,* drought*,* and their combinatory effect on the assembly of the microbiomes of oak phyllosphere and rhizosphere with respect to microbial richness*,* diversity*,* abundance*,* and community composition?* We hypothesized bacteria and fungi would respond differently to changing climate conditions, where fungi are expected to be more affected by drought and bacteria will respond more strongly to higher temperatures. Bacteria are associated to seeds and seed dormancy and therefore, probably more adapted to dryer conditions. Additionally, we hypothesize that analyzing the combinatory effect of warming and drought will demonstrate a different picture on microbial responses. We further hypothesize that the niches available for microbes will be colonized by similar species number, but different communities dependent on which climate factors they are exposed to.

**Table 1:** Overview of experimental studies that have investigated the effect of warming and/or drought on the bacterial and fungal community associated with plants

Study	Plant species and studied tissue	Type and total duration of warming/drought	Community metrics	Effect of warming	Effect of drought	Interactive effect of warming and drought
(Bálint et al. 2015)	*Populus balsamifera* (**Phyllosphere**)	Passive warming via open-top chambers; 6 months (May – Sep) over two years (2010/11)	**Fungal** Diversity, evenness and community composition	Decreased diversity, species richness and evenness; Changed community composition	Not tested	Not tested
(Kazenel et al. 2019)	Perennial grasses: Achnatherum lettermanii, Festuca thurberi, Poa pratensis (**Phyllosphere and roots**)	Infrared heating lamps; 23 years	**Fungal** diversity and community composition	No effect of warming on fungal diversity or community composition	Not tested	Not tested
(Firrincieli et al. 2020)	Wild Poplar *Populus trichocarpa* (**Phyllosphere**)	Based on climate conditions	**Bacterial** diversity	Not tested	Hot temperature reduced diversity	Not tested
(Wipf et al. 2021)	*Sorghum bicolor* (**Roots**)	Growth chamber, 7 days under stress	**Bacterial** species richness, community composition	Bacterial diversity increased	Bacterial diversity decreased	Changes in relative bacterial community composition
(Faticov et al. 2021)	*Quercus robur* (**Phyllosphere**)	Temperature increase of 2 °C over whole season	**Fungal** species richness, evenness, diversity	Fungal species richness & evenness decreased, changed community composition	Not tested	Not tested
(Fu et al. 2022)	Grassland of Inner Mongolia (**Roots**)	Intense drought and chronic drought	AM **fungal** richness, community composition, relative abundance diversity, inverse Simpson index	Not tested	Decreased AM fungal richness after three years continuous drought	Not tested
(Debray et al. 2022)	*Solanum lycopersicum* (**Phyllosphere**)	50% water deficit after 22d	**Bacterial and** fungal species richness, diversity, community composition	Not tested	Decreased bacterial species richness and diversity; increased fungal diversity	Not tested

To this end, we designed a multifactorial experimental set up on oak seedling that were grown in replicated climate chambers to investigate the effect of changing climatic conditions with focus on the combinatory effect of increased temperature and reduced soil moisture on the bacterial and fungal communities in phyllosphere and rhizosphere.

## Materials and methods

### Experimental setup and sampling

Acorns of the pedunculate oak (*Quercus robur L.*) were collected from a single tree in Stockholm to minimize the influence of oak genetic variation on the microbial community. Acorns were collected directly from the tree canopy before falling onto the ground to minimize the risk of microbial contamination from the environment, especially soil. The collected acorns were first rinsed in sterile Milli-Q water, then surface sterilized using sodium hypochlorite 10% for 30 min, followed by three rinses in sterile water, 5 min each. Surface sterilized acorns were stored in sterilized filter sand (0.4–0.8 mm) at 4 °C until use. The used filter sand was first autoclaved twice at 121 °C for 15 min with 24 h in between, then wetted with sterile Milli-Q to reach 30% soil moisture. The acorns were germinated at room temperature to a root length of 15 cm before being transferred to microcosms. The pots were filled with 73 ± 2 g of potting soil. Half of the samples were grown on soil with a low moisture (drought treatment, 15% soil moisture; soil directly from bag), the other half on soil with high moisture content (control treatment, 60% soil moisture; 60 mL water added to each microcosm). After the transfer of the germinated acorns to the microcosms, they were placed in climate boxes kept at specific temperatures (15 °C, 20 °C and 25 °C). The boxes constituted a controlled environment for the growth of the seedlings (Figure S1). There were three boxes for each temperature to minimize the effect of the boxes themselves, with the drought and control moisture samples being randomly distributed between those to mitigate any effect of the position of the samples within the boxes. In total 30 replicates per treatment combination were used. After being planted, the seedlings were left to grow until they reached a height of at least 8 cm, at which point the seedlings growing at 25 °C were sampled. One week after, seedlings grown at 20 °C were sampled, and the plants grown at 15 °C were sampled a month later. The difference in the sampling points was due to the effect of temperature on seedling growth. The leaves and the roots (with adhering soil) were sampled separately and transferred to 50 ml Sarstedt tubes. As two tissues were investigated per treatment, this made a total of 360 samples (3 temperatures x 2 soil moisture levels x 2 tissue types x 30 replicates).

### Sequencing of the fungal and bacterial microbiome

After sampling, the leaves and roots were lyophilized in the ScanVac CoolSafe™ (LaboGene, Allerød, Denkmark) and transferred to 1.5 ml Eppendorf tubes. Then, 7 small glass beads were added to each tube with the lyophilized samples and the samples were homogenized with a FastPrep Instrument (MP Biomedicals, Illkirch, France) for 60 s at 6 m/s. 30 mg (+-2 mg) of the material were used for DNA extraction using the FastDNA SPIN Kit for Soil (MP Biomedicals, Solon, OH, United States) according to the manufacturer’s instructions.

Polymerase chain reactions (PCRs) were conducted to amplify specific regions of the DNA to generate amplicon libraries. To investigate the bacterial communities, the variable region V4 of the 16S rRNA gene was amplified using universal primers and a standard PCR protocol. The primers 515f (5’-GTGCCAGCMGCCGCGGTAA-3’) and 806r (5’-GGACTACHVGGGTWTCTAAT-3’) [19] were used, being differently barcoded for each sample. To minimize amplification of host plastid and mitochondrial 16S rRNA regions, peptide nucleic acid (PNA) clamps were added to the reaction [42]. The PCR was performed in a total volume of 30 µl [5× Taq&Go (MP Biomedicals, Illkirch, France), 0.45 µl mPNA (5 µM), 0.45 µl pPNA (5 µM), 1.2 µl of each primer (5 µM), 19.7 µl PCR-grade water, and 1 µl template DNA] under the following cycling conditions: 95 °C for 5 min, 30 cycles of 95 °C for 30 s, 78 °C for 5 s, 54 °C for 30 s, 74 °C for 30 s and a final elongation at 74 °C for 5 min.

For the fungal community, an amplicon library of the ITS1 region was generated using the primers ITS5 [68] - ITS86R [66]. The sequence of the forward primer, including the pad sequence (ITS5_pad) was: 5′-TATGGTAATTGTGGAAGTAAAAGTCGTAACAAGG-3’, while the sequence including the pad sequence for the reverse primer (ITS86R_pad) was: 5′-AGTCAGCCAGGGTTCAAAGATTCGATGATTCAC-3′. The reaction was done in 10 µl [5 x Taq&Go, 1.2 µl MgCl_2_ (25 mM), 0.1 µl of each primer (10 µM), 5.6 µl PCR-grade water, and 1 µl template DNA] with the following cycling condition: 95 °C for 5 min, 35 cycles of 95 °C for 30 s, 58 °C for 35 s, 72 °C for 40 s, and a final elongation at 72 °C for 10 min. A second PCR step was performed to add barcoded primers for each sample, which was performed in 30 µl [5 x Taq&Go, 1.2 µl of each primer (5 µM), 19.6 µl PCR-grade water, and 2 µl template DNA from the previous step] with the following cycling conditions: 95 °C for 5 min, 15 cycles of 95 °C for 30 s, 53 °C for 30 s, 72 °C for 30 s and a final elongation at 72 °C for 5 min.

The products of the 16S rRNA amplification were then purified using the Wizard SV Gel and PCR Clean-Up System (Promega, Madison, WI, United States). The samples were pooled to equimolarity separately for rhizosphere and phyllosphere after measuring the concentrations with Nanodrop 2000. The PCR products of the ITS amplification were purified using AMPure XP beads (Beckman Coulter, Indianapolis, IN, United States) with a ratio of 1.8 µl magnet beads to 1 µl of PCR product.

All amplicons were sequenced using Illumina MiSeq V3 (2 × 300 bp, Illumina Inc., San Diego, CA, USA) chemistry.

Raw sequence data was demultiplexed using the cutadapt protocol [44], not allowing any mismatches or indels. Untrimmed sequences were discarded. The demultiplexed data was imported to QIIME 2 [13] for further processing. DADA2 [16] was used for sequence quality control, truncating both reverse and forward sequences at 170 bp and discarding chimeric sequences for the 16S rRNA library. For the ITS library, both forward and reverse sequences were truncated at 200 bp. Then, taxonomy was assigned to the representative sequences using the BLAST feature classifier [2] and the reference database SILVA v138 [53] for bacteria. Sequences assigned to host mitochondria and chloroplasts were removed from the feature table. For the fungal taxonomy assignment, the BLAST feature classifier and the reference data base UNITE V 8.3 released on 10.05.2021 [1] was used. Sequences assigned to the host were removed from the feature table. The taxonomy was then added to each biom file, which was imported to R [22] for analysis.

### Quantification of 16S rRNA and ITS gene copy numbers

For quantification of bacterial 16S and fungal ITS gene copy numbers in phyllosphere and rhizosphere, a quantitative real-time PCR (qPCR) was performed using the following primer pairs: 515f–806r for bacteria and ITS1–ITS2 [68] for fungi. Quantification of fluorescence was done in a Rotor-Gene 6000 real-time rotary analyser (Corbett Research, Sydney, Australia). The reaction mix contained 5 µl KAPA SYBR Green, 0.5 µl of each primer (10 µM each), 1 µl template DNA (diluted 1:100 in PCR grade water) and PCR-grade water to reach a reaction volume of 10 µl. mPNAs and pPNAs (5 µM each) were added as well for the 16S rRNA gene amplification. The following cycling conditions were used for the amplification of the 16S rRNA gene: 95 °C for 5 min, 40 cycles of 95 °C for 20 s, 78 °C for 5 s, 54 °C for 15 s and 72 °C for 30 s and a final melt curve of 72 °C to 95 °C. The amplification of the ITS1 gene for quantification was done using following cycling conditions: 95 °C for 3 min, 40 cycles of 95 °C for 5 s, 58 °C for 35 s and 72 °C for 5 s and a final melt curve of 72 °C to 95 °C. For each treatment and tissue, 10 were analyzed in triplicates, resulting in a total of 120 samples. The gene copy numbers found in negative controls were subtracted from the respective samples.

### Statistical analyses

Bacterial and fungal species richness (number of ASVs) and Shannon diversity were calculated from the rarefied amplicon dataset, while absolute microbial abundances were estimated via qPCR targeting the 16S rRNA and ITS gene copy numbers. Rarefaction was done to 300 and 500 sequences per sample for bacteria in phyllosphere and rhizosphere, respectively, and to 900 and 1000 sequences for fungi in phyllosphere and rhizosphere, respectively.

Shannon diversity, species richness and absolute abundance were modelled independently as a function of the fixed effects temperature, drought and their combinatory effect and the random effect of box number using linear mixed effect models with the function ‘lmer’ from the package lme4 [10]. To test for significance, the function ‘Anova’ from the car package (Fox und Weisberg 2019) was used. Marginal R-squared values were calculated for each fixed effect using the function ‘r.squaredGLMM’ from the MuMIn [36]. Pairwise comparisons of the different treatments were done using the lsmeans package [40]. The results were plotted using the ‘plot_richness’ function in phyloseq and the package ggplot2 [69]. To investigate the effect of the treatments on the bacterial and fungal community composition, the sequences were first normalized by cumulative sum scaling (CSS). The bacterial and fungal community composition were modeled as a function of temperature, drought and their combinatory effect using a PERMANOVA as applied to the function ‘adonis2’ in the vegan package [48] with 999 permutations.

Since PERMANOVA analysis does not distinguish the mechanism by which the community has changed i.e., species replacement (turnover) and species loss or gain (nestedness), we used the function ‘beta.multi’ from the package betapart [9] for beta partitioning. In beta partitioning, species turnover refers to the replacement of an existing species with a new species. In contrast, species nestedness refers to changes in the number of species, which can involve species gain or loss. Since this experiment was executed in a closed system, where the introduction of new species is unlikely, species turnover would imply that the changes incurred by one of our treatments may have led to an increase in abundance of a microbial species that was otherwise below detection limit. In this analysis, the changes in community composition based on the different treatments were analyzed by computing dissimilarity values between the seedlings grown at the different temperature levels (comparing 15 °C to 20 °C, 15 °C to 25 °C, and 20 °C to 25 °C). We also computed dissimilarity index for samplings subjected to drought and control treatments at each of the three temperature levels. The total β-diversity (Jaccard dissimilarity calculated based on presence–absence data) was partitioned into two indices, where βJTU is the turnover component of Jaccard dissimilarity and βJNE is the species nestedness component of the Jaccard dissimilarity.

To investigate which phyla explained the differences across the temperature levels and between drought and control treatments, differential abundance analysis was conducted using the linear discriminant analysis (LDA) effect size (LEfSe) algorithm incorporated in the ‘ldamarker’ function from the package microbial (v0.0.22) [35], after normalizing the data with CSS using the function ‘norm’ from the package microbiomeMarker [72]. The p-value cut-off was set to be below 0.05 for the Kruskal–Wallis tests and LDA scores > 2 were taken into consideration.

## Results

### The effect of warming and drought on the bacterial and fungal species richness, diversity, community composition and abundance in the rhizosphere and phyllosphere

**Fig. 1:**
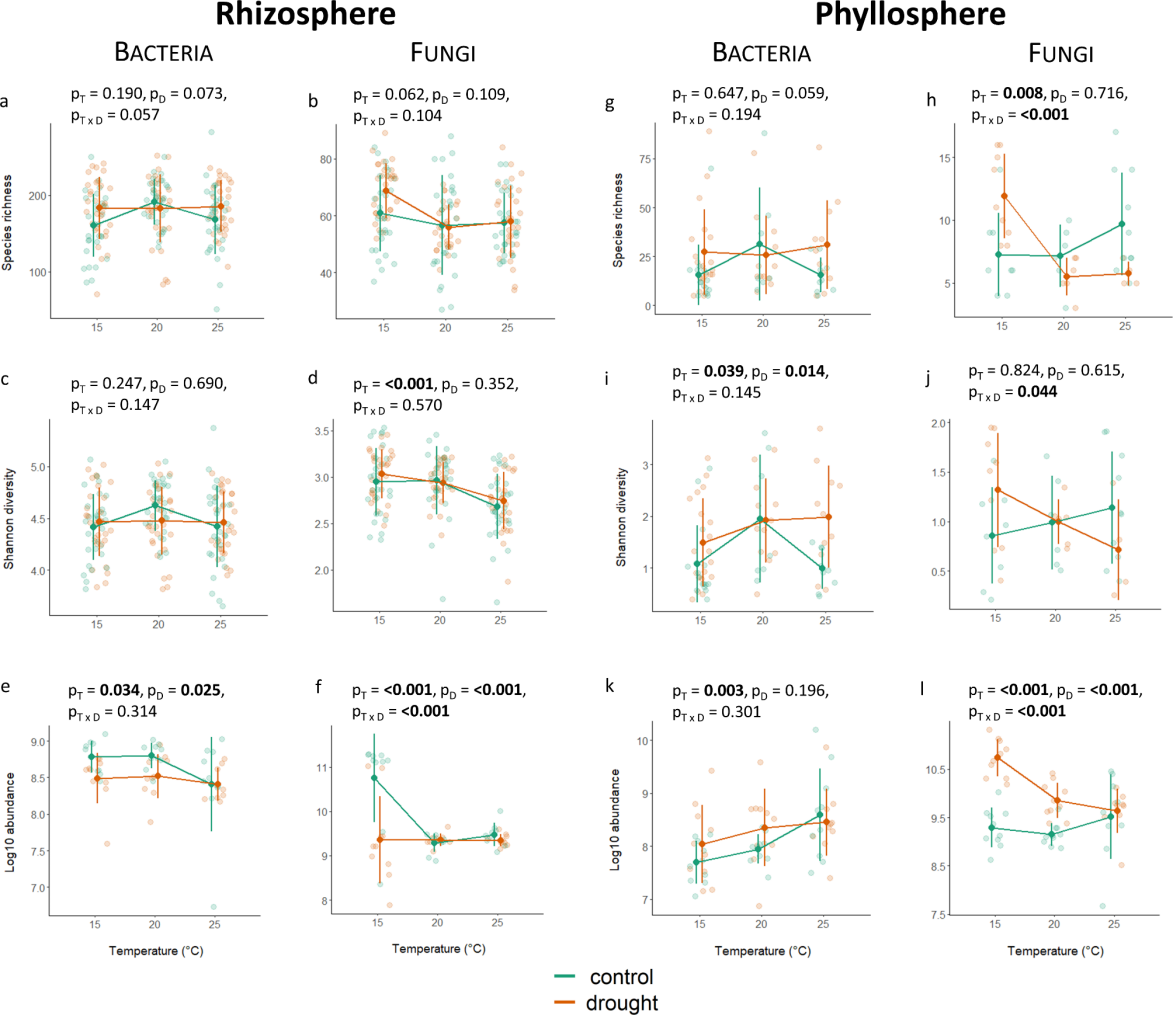
The impact of temperature and drought on the bacterial and fungal species richness (**a**, **b**, **g**, **h**), Shannon Index (**c**, **d**, **i**, **j**) and abundance (**e**, **f**, **k**, **l**) on rhizosphere (left) and phyllosphere (right) of common oak. Changes along the temperature gradient for control (green line) and drought (orange line) soil moisture samples are indicated. The dark circles represent the mean values and the error bars represent standard deviations. Raw data points (bright circles) are horizontally jittered to avoid overlap. T: temperature; D: drought, T x D: combinatory effect of temperature and drought

Bacterial and fungal species richness i.e., number of ASVs, in the rhizosphere, as well as the bacterial diversity, were not affected by temperature, drought or their combinatory effect. However, fungal diversity was influenced by temperature and was the lowest at 25 °C (Fig. 1a, b, c and d and Table S1). Bacterial abundance in the rhizosphere was significantly higher in control than in drought at 15 °C and 20 °C, but at 25 °C it decreased to the same level observed in drought. The fungal abundance was affected by temperature, drought and their combinatory effect, with a particularly high fungal abundance in control at 15 °C (Fig. 1e and f and Table S1).

In the phyllosphere, bacterial species richness was not significantly affected by temperature, drought or their combinatory effect. Fungal species richness in phyllosphere was affected by temperature and the combinatory effect of temperature and drought, while species richness decreased with higher temperature in drought but increased with temperature in control (Fig. 1g and h and Table S1). The bacterial diversity in the phyllosphere was affected by temperature and drought; in drought, bacterial diversity increased with temperature. Here, fungal diversity decreased with temperature in drought, but increased with temperature in control (Fig. 1i and j and Table S1). Bacterial abundance in phyllosphere was affected only by temperature and increased with temperature for both drought and control, whereas fungal abundance was affected by temperature, drought and their combinatory effect. Fungal abundance decreased with temperature in drought and increased with temperature in control (Fig. 1k and l and Table S1).

**Fig. 2:**
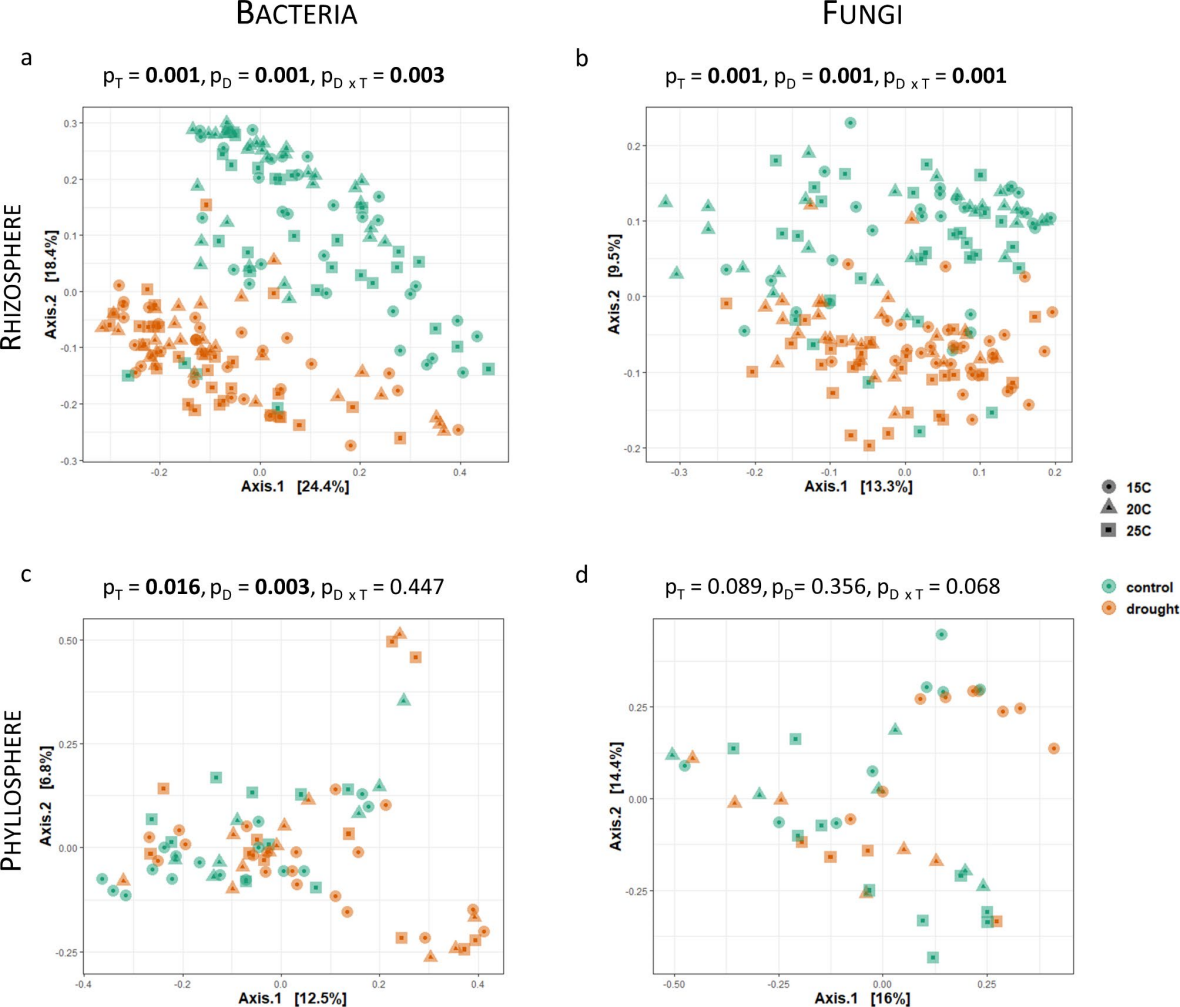
Impact of temperature on the bacterial (left) and fungal (right) community composition on oak rhizosphere (**a**, **b**) and phyllosphere (**c**, **d**), visualized using principle coordinate analysis (PCoA) based on Bray-Curtis dissimilarities. P-values and R^2^-values were calculated using PERMANOVA (absolute count data; function ´adonis2´ from the package vegan). Significant values are indicated in bold. T: temperature; D: drought, D x T: combinatory effect of drought and temperature

The community composition of both bacteria and fungi in the rhizosphere were affected by temperature and drought and their combinatory effect (Fig. 2a and b and Table S1). In the phyllosphere, however, only the bacterial composition was affected by temperature and drought, whereas no effect was observed for the fungal community due to temperature and drought or their combinatory effect (Fig. 2c and d).

In general, fungi exhibit a more pronounced response to climate factors compared to bacteria in terms of diversity, richness, and abundance, within both the rhizosphere and the phyllosphere. Regarding the community composition, both bacteria and fungi exhibit significant responses in the rhizosphere, while the communities in the phyllosphere remain unaffected.

### The response of specific taxonomic groups to warming and drought

**Fig. 3:**
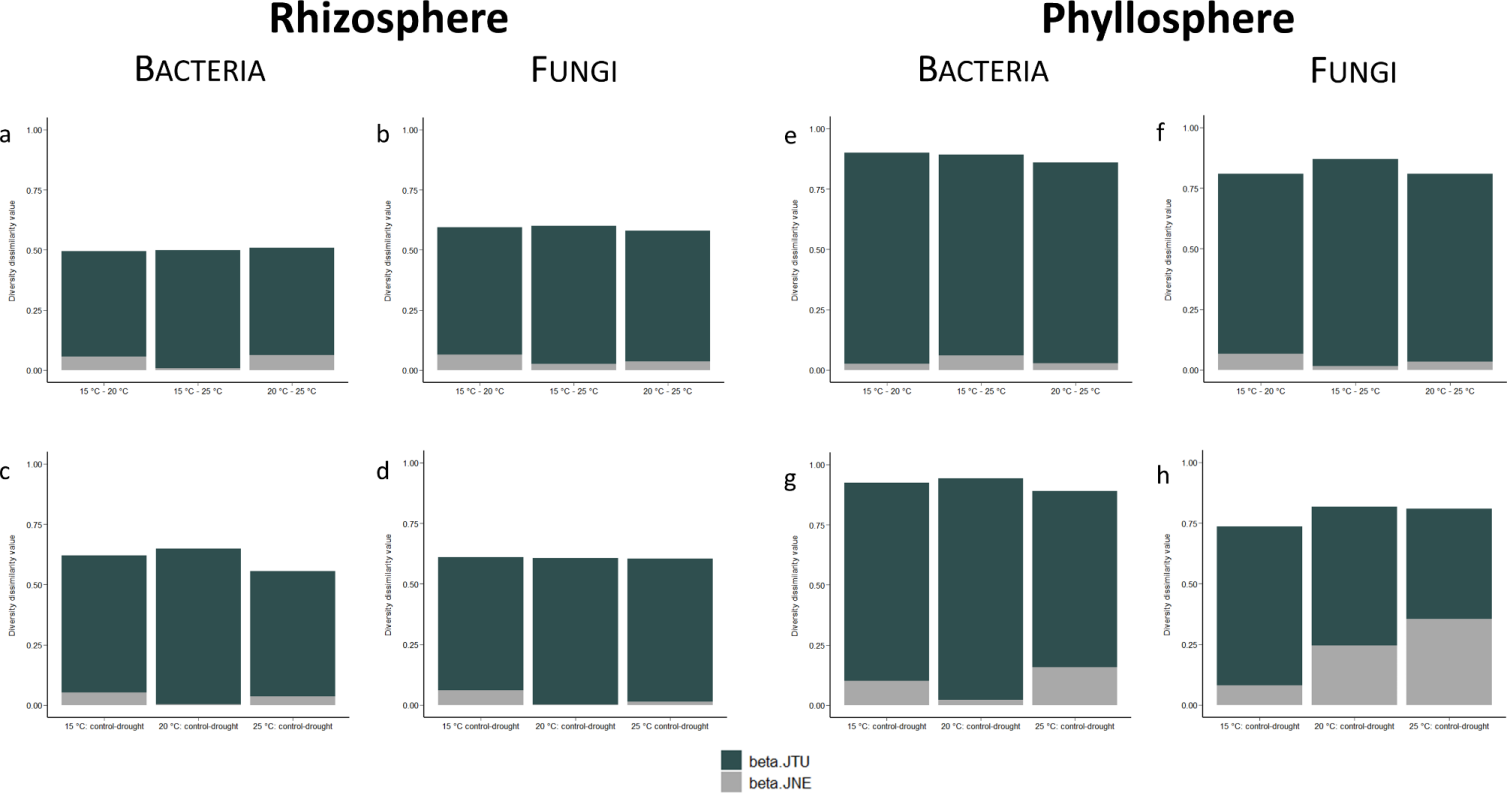
Partitioning of the total β-diversity (Jaccard dissimilarity index) between the different temperature treatments (**a**, **b**, **e**, **f**) and the different soil moisture treatments at the specific temperatures (**c**, **d**, **g**, **h**) into the components of species turnover (beta.JTU) and change in species numbers (beta.JNE) for the rhizosphere (left) and phyllosphere (right) communities of bacteria and fungi

Control conditions in rhizosphere enriched the bacterial phyla *Acidobacteriota*,* Firmicutes*,* Planctomycetota* and *Proteobacteria* at 20 °C, while *Firmicutes* enriched also at 25 °C (Fig. 3a). Regarding fungi, *Basidiomycota* and *Mortierellomycota* enriched at 15 °C, an unassigned phylum enriched at 20 °C and *Ascomycota*, *Basidiomycota* and another unassigned phylum enriched at 25 °C (Fig. 3b).

**Fig. 4:**
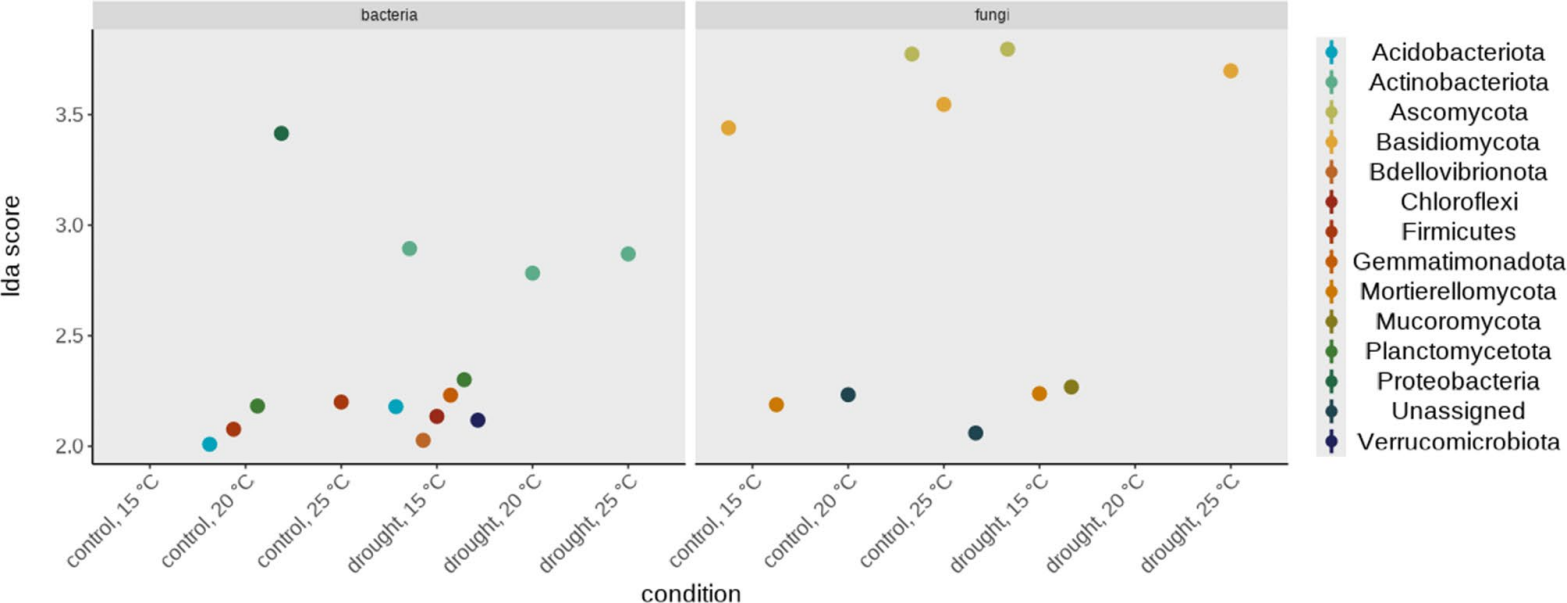
Differential abundance analysis of bacterial (**a**) and fungal (**b**) taxa under different growth conditions (temperature: 15 °C, 20 °C, 25 °C; soil moisture: control, drought) in rhizosphere using the linear discriminant analysis (LDA) effect size (LEfSe) algorithm. LDA-scores were calculated using the run_lefse function form the package microbiomeMarker with p-value cut-off set to below 0.0.5 for Kruskal-Wallis tests and we considered only LDA scores > 2

Drought conditions in rhizosphere enriched the bacterial taxa *Acidobacteriota*, *Actinobacteriota*, *Bdellovibrionota*, *Chloroflexi*, *Gemmatimonadota*, *Planctomycetota* and *Verrucomicrobiota* at 15 °C and *Actinobacteriota* at 20 °C and 25 °C, respectively (Fig. 4a). The fungal taxa *Ascomycota*, *Mortierellomycota* and Mucoromycota enriched at 15 °C and Basidiomycota at 25 °C (Fig. 4b).

In the phyllosphere, we did not observe any taxonomic shifts at any soil moisture nor temperature levels.

Comparing drought and control conditions we found that the bacterial taxa *Actinobacteriota* enriched only in drought at all temperature levels and *Firmicutes* enriched only in the control treatment at higher temperatures. Overall, we can state that the response of rhizosphere bacteria and fungi to increasing temperatures is influenced by the levels of soil moisture to which they are subjected.

### Species turnover vs. species nestedness

The shift in bacterial and fungal community composition in rhizosphere between different temperatures (15–20 °C, 15–25 °C, 20–25 °C) was mainly explained by species turnover (βJTU) and only to a marginal extent by species nestedness (βJNE) (Fig. 3a and b and Table S2). Similarly, the shift in bacterial and fungal community composition in rhizosphere between drought and control at specific temperatures (15 °C control – drought, 20 °C control – drought, 25 °C control – drought) was explained by species turnover and only to a minor extent by a change in species numbers (Fig. 3c and d and Table S2).

The shift in bacterial community composition in phyllosphere between different temperatures (15–20 °C, 15–25 °C, 20–25 °C) was primarily explained by species turnover (βJTU) and only to a marginal extent by species nestedness (βJNE), whereas temperature showed no effect on the fungal community composition (Fig. 3e and f and Table S2). The shift in bacterial community composition in phyllosphere between drought and control at specific temperatures (15 °C control – drought, 20 °C control – drought, 25 °C control – drought) was mainly explained by species turnover, with changes in species number playing a lesser role (Fig. 3g and Table S2). For the fungal community composition in phyllosphere a larger part of the shift was due to changes in species number, especially at higher temperatures (Fig. 3h and Table S2).

In summary, those data indicate that warming and drought determines which microbes present in seeds colonize the rhizosphere and phyllosphere of the developing plant, and that the composition of microbial species is more impacted by these environmental factors than the overall number of species.

## Discussion

In the present study we tested the effect of temperature, drought and their combinatory effect on the assembly of the fungal and bacterial communities in oak rhizosphere and phyllosphere from seed to seedling stage. The combinatory effect of temperature and drought had an impact on fungal diversity in the phyllosphere, as well as fungal abundance in both the rhizosphere and phyllosphere. Additionally, the combinatory effect significantly influenced the bacterial and fungal communities in rhizosphere, but not in the phyllosphere. It was observed that temperature and drought jointly affected the composition of the rhizosphere community. These results highlight the importance of considering the combinatory effects of temperature and drought in understanding the dynamics and help us predict the responses of microbial communities to climate change.

Our findings indicate that temperature had a more pronounced effect on fungal diversity compared to bacterial diversity, and was more evident in the phyllosphere than the rhizosphere. The more evident effect in the phyllosphere than the rhizosphere could be due to the inherit differences in these habitats. The phyllosphere is directly exposed to the increasing temperature, while the soil may act as a buffer, delaying the effect of increasing temperature on the rhizosphere. Further, the phyllosphere is known to be a more fluctuating and harsh environment compared to the rhizosphere with wild variations in temperatures, UV and moisture levels, which prevents the majority of microbiological organisms from growing [39]. For instance, previous studies have found that temperature had a significant negative effect on the diversity of the phyllosphere microbiome and no effect on soil [7, 31, 56]. Furthermore, we found that temperature had a contrasting effect on the fungal diversity in phyllosphere where it decreased significantly under drought condition and increased significantly in the control treatment. This contrasting effect could be explained by the fact that warmer temperatures (20–30 °C) are optimal for the growth of the majority of fungal species which could result in an increase in the number of observed species. However, considering that water is a main driver of fungal growth and survival [18, 33, 41], the mere lack of water can have detrimental effect, which are further exacerbated by elevated temperatures, even though some fungi have traits, which can mitigate drought, including thick cell walls, osmolytes and melanin [60, 65]. In fact, we found that fungal abundance follows a similar pattern where it was significantly reduced with increasing temperature in the drought compared to the control treatment.

Even though temperature did not affect the number of observed species in the rhizosphere, it had a strong effect on the fungal community composition. This result, however, is not surprising considering that beta partitioning analysis showed that the change in community composition was primarily driven by species turnover. The analysis indicates that the major change in community composition is primarily due to species turnover, meaning that new species are replacing existing ones in the community. In this context, varying temperatures can result in a microbial turnover due to changes in environmental conditions [38]. Additionally, temperature can have a direct impact on plant growth, leading to alterations in root exudate, hence, shaping the selection and composition of the rhizosphere community [64]. Our findings contrast with those of Kazenel et al. ’s [37], who did not observe any changes in community composition with temperature in rhizosphere and phyllosphere. Differences in results may originate from different plant species studied and extremely differing experimental designs, as their experiment included experimental warming over 23 years in natural conditions. Conversely, Zhang et al. [74] demonstrated a significant influence of temperature on the bacterial community composition in the rice rhizosphere.

Temperature has a negative effect on the bacterial and fungal abundance in the rhizosphere and in the phyllosphere, except in phyllosphere control conditions where it increases. As mentioned above, elevated temperature can have a positive effect on plant growth, which can result in increased competition with soil microorganisms over resources (e.g. water and nutrients), limiting both fungal and bacterial growth in the soil. Elevated temperature is also associated with increased transpiration [58] which may relocate these resources from below to above ground, providing phyllosphere associated microorganisms with more resources. This hypothesis is supported by the fact that we only observe this in control conditions where water is available, and not in drought conditions. Although drought had a weak effect on microbial diversity, its impact was profound on community composition. Similarly as the effect of temperature, the influence of drought on community composition was primarily due to species turnover rather than changes in species number. The change in community composition was, expectedly, more evident in the rhizosphere than phyllosphere. As anticipated, *Actinobacteriota* was significantly enriched at all temperature levels only in drought conditions. The beneficial effects to plants during drought has detailed been discussed in Ebrahimi-Zarandi et al. [26], e.g. *Actinobacteriota* can help plants in nutrient uptake, phosphate and potassium solubilization, siderophore production and nitrogen fixation [15, 29, 45, 49]. Drought has been shown to have several effects on plants, ranging from root traits [55], decreased root exudation [62], and immune response [20, 46, 52]. Drought can also have an effect on soil structure (e.g. soil aggregates), and chemistry (e.g. carbon content, nutrient solubility, and pH), all of which can influence the structure and function of the soil microbiome. For example, drought can increase microaggregates resulting in the formation of smaller habitats and diversity islands, as well as effecting community network [23, 63].

The rhizosphere and the phyllosphere showed contrasting responses to drought, e.g., lower fungal abundance in rhizosphere and higher fungal abundance in phyllosphere. This effect was mostly evident at lower temperature (15 °C) and tended to diminish with warming. Certain species may produce a higher number of spores during drought conditions, which can complicate the interpretation of our results. Sporulation events could potentially skew fungal abundance measurements, leading to an overestimation of habitat colonization for fungi.

## Conclusion

The framework presented in this study captures the main changes in bacterial and fungal diversity, composition and abundance due to climatic impacts and provides insights into the effects of climate change on the oak seedlings’ microbiome. Although, the effect of drought was not very evident, there was a clear effect on community composition and abundance. Therefore, it should be avoided to rely on diversity indicators as the sole indicator to measure the effect of climate change. Since most of the change we observed in community composition was due to community turnover, we expect a drastic change in functional diversity. Therefore, for future direction, it will be important to conduct a similar study to implement multi-omics techniques. Furthermore, it was difficult to find a unifying effect of temperature, even though the effect of temperature was more evident on phyllosphere, where it reduced fungal diversity and abundance. However, this effect was only under drought conditions and opposite in control conditions. For this reason we suggest future studies refrain from making generalized statements about the effect of climate change on the plant microbiome without considering these aspects. The change in microbial community composition and enrichment of *Actinobacteriota* during drought suggests an adaptation and – more importantly – a selection of rhizosphere microbes. We do note that although this study was performed under controlled conditions in climate chambers, and it is expected that some aspects may differ in natural ecosystems, we hope it can lay the ground for future research. In conclusion, it is essential to understand the impact of climatic changes on the plant microbiome and plant-microbe interactions to preserve the health of forests and their ecosystems.

### Electronic supplementary material

Below is the link to the electronic supplementary material.

Supplementary Material 1

## Data Availability

The dataset supporting the conclusions of this article will be available in the European Nucleotide Archive (ENA) repository with the accession number PRJNA1082614 upon acceptance of the manuscript. The code used in this study will be provided on Zenodo upon acceptance of the manuscript.

## References

[1] Abarenkov K, Henrik Nilsson R, Larsson K-H, Alexander IJ, Eberhardt U, Erland S, Høiland K, Kjøller R, Larsson E, Pennanen T, Sen R, Taylor AFS, Tedersoo L, Ursing BM, Vrålstad T, Liimatainen K, Peintner U, Kõljalg U. The UNITE database for molecular identification of fungi–recent updates and future perspectives. New Phytol. 2010;186:2, 281–5. 20409185 10.1111/j.1469-8137.2009.03160.x

[2] Altschul SF, Gish W, Miller W, Myers EW, Lipman DJ. Basic local alignment search tool. J Mol Biol. 1990;215:3, 403–10. 2231712 10.1016/S0022-2836(05)80360-2

[3] Andreo-Jimenez B, Vandenkoornhuyse P, Lê Van A, Heutinck A, Duhamel M, Kadam N, Jagadish K, Ruyter-Spira C, Bouwmeester H. 2019. Plant host and drought shape the root associated fungal microbiota in rice. PeerJ 7, e7463. 10.7717/peerj.7463 PMC6744933 31565550

[4] Arneth A, Shin Y-J, Leadley P, Rondinini C, Bukvareva E, Kolb M, Midgley GF, Oberdorff T, Palomo I, Saito O. Post-2020 biodiversity targets need to embrace climate change. Proc Natl Acad Sci USA. 2020;117:49, 30882–91. 33288709 10.1073/pnas.2009584117 PMC7739876

[5] Aydogan EL, Moser G, Müller C, Kämpfer P, Glaeser SP. Long-term warming shifts the composition of bacterial communities in the Phyllosphere of Galium album in a Permanent Grassland Field-Experiment. Front Microbiol. 2018;9:144. 29487575 10.3389/fmicb.2018.00144 PMC5816784

[6] Bahram M, Hildebrand F, Forslund SK, Anderson JL, Soudzilovskaia NA, Bodegom PM, Bengtsson-Palme J, Anslan S, Coelho LP, Harend H, Huerta-Cepas J, Medema MH, Maltz MR, Mundra S, Olsson PA, Pent M, Põlme S, Sunagawa S, Ryberg M, Tedersoo L, Bork P. Structure and function of the global topsoil microbiome. Nature. 2018;560:7717, 233–7. 30069051 10.1038/s41586-018-0386-6

[7] Bálint M, Bartha L, O’Hara RB, Olson MS, Otte J, Pfenninger M, Robertson AL, Tiffin P, Schmitt I. Relocation, high-latitude warming and host genetic identity shape the foliar fungal microbiome of poplars. Mol Ecol. 2015;24:1, 235–48. 25443313 10.1111/mec.13018

[8] Barnard RL, Osborne CA, Firestone MK. Responses of soil bacterial and fungal communities to extreme desiccation and rewetting. ISME J. 2013;7:11, 2229–41. 23823489 10.1038/ismej.2013.104 PMC3806258

[9] Baselga A, Orme D, Villeger S, Bortoli JD, Leprieur F. Maxime Logez, Sara Martinez-Santalla, Ramiro Martin-Devasa, Carola Gomez-Rodriguez, and Rosa M. Crujeiras. 2012. betapart: Partitioning Beta Diversity into Turnover and Nestedness Components.

[10] Bates D, Mächler M, Bolker B, Walker S. Fitting Linear mixed-effects models using lme4. J Stat Soft. 2015;67:1. 10.18637/jss.v067.i01

[11] Bechtold EK, Ryan S, Moughan SE, Ranjan R, Nüsslein K. 2021. Phyllosphere Community Assembly and Response to Drought stress on common tropical and temperate forage grasses. Appl Environ Microbiol 87, 17, e0089521. 10.1128/AEM.00895-21 PMC8357280 34161142

[12] Bellard C, Bertelsmeier C, Leadley P, Thuiller W, Courchamp F. Impacts of climate change on the future of biodiversity. Ecol Lett. 2012;15:4, 365–77. 22257223 10.1111/j.1461-0248.2011.01736.x PMC3880584

[13] Bolyen E, Rideout JR, Dillon MR, Bokulich NA, Abnet CC, Al-Ghalith GA, Alexander H, Alm EJ, Arumugam M, Asnicar F, Bai Y, Bisanz JE, Bittinger K, Brejnrod A, Brislawn CJ, Brown CT, Callahan BJ, Caraballo-Rodríguez AM, Chase J, Cope EK, Da Silva R, Diener C, Dorrestein PC, Douglas GM, Durall DM, Duvallet C, Edwardson CF, Ernst M, Estaki M, Fouquier J, Gauglitz JM, Gibbons SM, Gibson DL, Gonzalez A, Gorlick K, Guo J, Hillmann B, Holmes S, Holste H, Huttenhower C, Huttley GA, Janssen S, Jarmusch AK, Jiang L, Kaehler BD, Kang KB, Keefe CR, Keim P, Kelley ST, Knights D, Koester I, Kosciolek T, Kreps J, Langille MGI, Lee J, Ley R, Liu Y-X, Loftfield E, Lozupone C, Maher M, Marotz C, Martin BD, McDonald D, McIver LJ, Melnik AV, Metcalf JL, Morgan SC, Morton JT, Naimey AT, Navas-Molina JA, Nothias LF, Orchanian SB, Pearson T, Peoples SL, Petras D, Preuss ML, Pruesse E, Rasmussen LB, Rivers A, Robeson MS, Rosenthal P, Segata N, Shaffer M, Shiffer A, Sinha R, Song SJ, Spear JR, Swafford AD, Thompson LR, Torres PJ, Trinh P, Tripathi A, Turnbaugh PJ, Ul-Hasan S, van der Hooft JJJ, Vargas F, Vázquez-Baeza Y, Vogtmann E, von Hippel M, Walters W, Wan Y, Wang M, Warren J, Weber KC, Williamson AD, Xu. Z. Z., Zaneveld, J. R., Zhang, Y., Zhu, Q., Knight, R., and Caporaso, J. G. 2019. Reproducible, interactive, scalable and extensible microbiome data science using QIIME 2. *Nat Biotechnol* 37, 8, 852–857. 10.1038/s41587-019-0209-9 PMC7015180 31341288

[14] Bouasria A, Mustafa T, de Bello F, Zinger L, Lemperiere G, Geremia RA, Choler P. Changes in root-associated microbial communities are determined by species-specific plant growth responses to stress and disturbance. Eur J Soil Biol. 2012;52:59–66. 10.1016/j.ejsobi.2012.06.003

[15] Boubekri K, Soumare A, Mardad I, Lyamlouli K, Ouhdouch Y, Hafidi M, Kouisni L. Multifunctional role of Actinobacteria in agricultural production sustainability: a review. Microbiol Res. 2022;261:127059. 35584559 10.1016/j.micres.2022.127059

[16] Callahan BJ, McMurdie PJ, Rosen MJ, Han AW, Johnson AJA, Holmes SP. DADA2: high-resolution sample inference from Illumina amplicon data. Nat Methods. 2016;13:7, 581–3. 27214047 10.1038/nmeth.3869 PMC4927377

[17] Campisano A, Albanese D, Yousaf S, Pancher M, Donati C, Pertot I. Temperature drives the assembly of endophytic communities’ seasonal succession. Environ Microbiol. 2017;19:8, 3353–64. 28654220 10.1111/1462-2920.13843

[18] Canini F, Zucconi L, Pacelli C, Selbmann L, Onofri S, Geml J. 2019. Vegetation, pH and water content as main factors for shaping fungal richness, Community Composition and Functional guilds distribution in soils of Western Greenland. Front Microbiol 10. 10.3389/fmicb.2019.02348 PMC6797927 31681213

[19] Caporaso JG, Kuczynski J, Stombaugh J, Bittinger K, Bushman FD, Costello EK, Fierer N, Peña AG, Goodrich JK, Gordon JI, Huttley GA, Kelley ST, Knights D, Koenig JE, Ley RE, Lozupone CA, McDonald D, Muegge BD, Pirrung M, Reeder J, Sevinsky JR, Turnbaugh PJ, Walters WA, Widmann J, Yatsunenko T, Zaneveld J, Knight R. QIIME allows analysis of high-throughput community sequencing data. Nat Methods. 2010;7:5, 335–6. 20383131 10.1038/nmeth.f.303 PMC3156573

[20] Cavicchioli R, Ripple WJ, Timmis KN, Azam F, Bakken LR, Baylis M, Behrenfeld MJ, Boetius A, Boyd PW, Classen AT, Crowther TW, Danovaro R, Foreman CM, Huisman J, Hutchins DA, Jansson JK, Karl DM, Koskella B, Welch M, Martiny DB, Moran JBH, Orphan MA, Reay VJ, Remais DS, Rich JV, Singh VI, Stein BK, Stewart LY, Sullivan FJ, van Oppen MB, Weaver MJH, Webb SC, E. A., and, Webster NS. Scientists’ warning to humanity: microorganisms and climate change. Nat Rev Microbiol. 2019;17:9, 569–86. 31213707 10.1038/s41579-019-0222-5 PMC7136171

[21] Chen Y, Yao Z, Sun Y, Wang E, Tian C, Sun Y, Liu J, Sun C, Tian L. Current studies of the effects of Drought stress on Root exudates and Rhizosphere microbiomes of Crop Plant species. Int J Mol Sci. 2022;23:4. 10.3390/ijms23042374 PMC8874553 35216487

[22] 2022. *R* Core Team. *A language and environment for statistical computing*. R Core Team.

[23] de Vries FT, Griffiths RI, Bailey M, Craig H, Girlanda M, Gweon HS, Hallin S, Kaisermann A, Keith AM, Kretzschmar M, Lemanceau P, Lumini E, Mason KE, Oliver A, Ostle N, Prosser JI, Thion C, Thomson B, Bardgett RD. Soil bacterial networks are less stable under drought than fungal networks. Nat Commun. 2018;9:1. 30072764 10.1038/s41467-018-05516-7 PMC6072794

[24] Debray R, Socolar Y, Kaulbach G, Guzman A, Hernandez CA, Curley R, Dhond A, Bowles T, Koskella B. Water stress and disruption of mycorrhizas induce parallel shifts in phyllosphere microbiome composition. New Phytol. 2022;234(6):2018–31. 34668201 10.1111/nph.17817

[25] Dewan S, de Frenne P, Leroux O, Nijs I, Mijnsbrugge V, K., and, Verheyen K. Phenology and growth of Fagus sylvatica and Quercus robur seedlings in response to temperature variation in the parental versus offspring generation. Plant Biol. 2020;22(Suppl 1):113–22. 30739399 10.1111/plb.12975

[26] Ebrahimi-Zarandi M, Etesami H, Glick BR. Fostering plant resilience to drought with Actinobacteria: unveiling perennial allies in drought stress tolerance. Plant Stress. 2023;10:100242. 10.1016/j.stress.2023.100242

[27] Edwards J, Johnson C, Santos-Medellín C, Lurie E, Podishetty NK, Bhatnagar S, Eisen JA, Sundaresan V. Structure, variation, and assembly of the root-associated microbiomes of rice. Proc Natl Acad Sci USA. 2015;112:8, E911–20. 25605935 10.1073/pnas.1414592112 PMC4345613

[28] Ercolani GL. Distribution of epiphytic bacteria on olive leaves and the influence of leaf age and sampling time. Microb Ecol. 1991;21(1):35–48. 24194200 10.1007/BF02539143

[29] Etesami H, Adl SM. 2020. Plant Growth-Promoting Rhizobacteria (PGPR) and Their Action Mechanisms in Availability of Nutrients to Plants. In *Phyto-Microbiome in Stress Regulation*, M. Kumar, V. Kumar and R. Prasad, Eds. Environmental and Microbial Biotechnology. Springer Singapore, Singapore, 147–203. DOI = 10.1007/978-981-15-2576-6_9.

[30] Fahad S, Sonmez O, Saud S, Wang D, Wu C, Adnan M, Turan V. *Climate change and plants*. *Biodiversity, Growth and interactions*. Milton: Footprints of Climate Variability on Plant Diversity Ser. Taylor & Francis Group; 2021.

[31] Faticov M, Abdelfattah A, Roslin T, Vacher C, Hambäck P, Blanchet FG, Lindahl BD, Tack AJM. Climate warming dominates over plant genotype in shaping the seasonal trajectory of foliar fungal communities on oak. New Phytol. 2021;231:5, 1770–83. 33960441 10.1111/nph.17434

[32] Fujimura KE, Egger KN, Henry GHR. The effect of experimental warming on the root-associated fungal community of Salix arctica. ISME J. 2008;2(1):105–14. 18180749 10.1038/ismej.2007.89

[33] Herman KC, Bleichrodt R. Go with the flow: mechanisms driving water transport during vegetative growth and fruiting. Fungal Biology Reviews. 2022;41:10–23. 10.1016/j.fbr.2021.10.002

[34] Jansson JK, Hofmockel KS. Soil microbiomes and climate change. Nat Rev Microbiol. 2020;18:1, 35–46. 31586158 10.1038/s41579-019-0265-7

[35] Kai Guo PG. 2021. *Microbial*. *Do 16s Data Analysis and Generate Figures*. Comprehensive R Archive Network (CRAN).

[36] Kamil Barton. MuMIn. Multi-Model Inference; 2009.

[37] Kazenel MR, Kivlin SN, Taylor DL, Lynn JS, Rudgers JA. Altitudinal gradients fail to predict fungal symbiont responses to warming. Ecology. 2019;100:8. e02740. 10.1002/ecy.2740 31006112

[38] Kelishadi H, Mosaddeghi MR, Ayoubi S, Mamedov AI. Effect of temperature on soil structural stability as characterized by high energy moisture characteristic method. CATENA. 2018;170:290–304. 10.1016/j.catena.2018.06.015

[39] Koskella B. The phyllosphere. Curr Biology: CB. 2020;30:19, R1143–6. 33022257 10.1016/j.cub.2020.07.037

[40] Lenth RV. Least-squares means: the R Package lsmeans. J Stat Soft. 2016;69:1, 1–33. 10.18637/jss.v069.i01

[41] Lew RR. How does a hypha grow? The biophysics of pressurized growth in fungi. Nat Rev Microbiol. 2011;9:7, 509–18. 21643041 10.1038/nrmicro2591

[42] Lundberg DS, Yourstone S, Mieczkowski P, Jones CD, Dangl JL. Practical innovations for high-throughput amplicon sequencing. Nat Methods. 2013;10:10, 999–1002. 23995388 10.1038/nmeth.2634

[43] Maestre FT, Delgado-Baquerizo M, Jeffries TC, Eldridge DJ, Ochoa V, Gozalo B, Quero JL, García-Gómez M, Gallardo A, Ulrich W, Bowker MA, Arredondo T, Barraza-Zepeda C, Bran D, Florentino A, Gaitán J, Gutiérrez JR, Huber-Sannwald E, Jankju M, Mau RL, Miriti M, Naseri K, Ospina A, Stavi I, Wang D, Woods NN, Yuan X, Zaady E, Singh BK. 2015. Increasing aridity reduces soil microbial diversity and abundance in global drylands. *Proceedings of the National Academy of Sciences of the United States of America* 112, 51, 15684–15689. 10.1073/pnas.1516684112 PMC4697385 26647180

[44] Martin M. Cutadapt removes adapter sequences from high-throughput sequencing reads. EMBnet j. 2011;17:1. 10.14806/ej.17.1.200

[45] Narsing Rao MP, Lohmaneeratana K, Bunyoo C, Thamchaipenet A. Actinobacteria-plant interactions in alleviating abiotic stress. Plants (Basel Switzerland). 2022;11:21. 10.3390/plants11212976 PMC9658302 36365429

[46] Naylor D, Coleman-Derr D. Drought stress and Root-Associated Bacterial communities. Front Plant Sci. 2017;8:2223. 29375600 10.3389/fpls.2017.02223 PMC5767233

[47] Naylor D, DeGraaf S, Purdom E, Coleman-Derr D. Drought and host selection influence bacterial community dynamics in the grass root microbiome. ISME J. 2017;11:12, 2691–704. 28753209 10.1038/ismej.2017.118 PMC5702725

[48] Oksanen et al. 2013.

[49] Oyedoh OP, Yang W, Dhanasekaran D, Santoyo G, Glick BR, Babalola OO. Rare rhizo-Actinomycetes: a new source of agroactive metabolites. Biotechnol Adv. 2023;67:108205. 37356598 10.1016/j.biotechadv.2023.108205

[50] Pecl GT, Araújo MB, Bell JD, Blanchard J, Bonebrake TC, Chen I-C, Clark TD, Colwell RK, Danielsen F, Evengård B, Falconi L, Ferrier S, Frusher S, Garcia RA, Griffis RB, Hobday AJ, Janion-Scheepers C, Jarzyna MA, Jennings S, Lenoir J, Linnetved HI, Martin VY, McCormack PC, McDonald J, Mitchell NJ, Mustonen T, Pandolfi JM, Pettorelli N, Popova E, Robinson SA, Scheffers BR, Shaw JD, Sorte CJB, Strugnell JM, Sunday JM, Tuanmu M-N, Vergés A, Villanueva C, Wernberg T, Wapstra E, Williams SE. 2017. Biodiversity redistribution under climate change: Impacts on ecosystems and human well-being. *Science (New York, N.Y.)* 355, 6332. 10.1126/science.aai9214 28360268

[51] Peñuelas J, Rico L, Ogaya R, Jump AS, Terradas J. Summer season and long-term drought increase the richness of bacteria and fungi in the foliar phyllosphere of Quercus ilex in a mixed Mediterranean forest. Plant Biol. 2012;14:4, 565–75. 22289059 10.1111/j.1438-8677.2011.00532.x

[52] Preece C, Verbruggen E, Liu L, Weedon JT, Peñuelas J. Effects of past and current drought on the composition and diversity of soil microbial communities. Soil Biol Biochem. 2019;131:28–39. 10.1016/j.soilbio.2018.12.022

[53] Quast C, Pruesse E, Yilmaz P, Gerken J, Schweer T, Yarza P, Peplies J, O. Glöckner F. The SILVA ribosomal RNA gene database project: improved data processing and web-based tools. Nucleic Acids Res. 2013;41(Database issue):D590–6. 23193283 10.1093/nar/gks1219 PMC3531112

[54] Radchuk V, Reed T, Teplitsky C, van de Pol M, Charmantier A, Hassall C, Adamík P, Adriaensen F, Ahola MP, Arcese P, Miguel Avilés J, Balbontin J, Berg KS, Borras A, Burthe S, Clobert J, Dehnhard N, de Lope F, Dhondt AA, Dingemanse NJ, Doi H, Eeva T, Fickel J, Filella I, Fossøy F, Goodenough AE, Hall SJG, Hansson B, Harris M, Hasselquist D, Hickler T, Joshi J, Kharouba H, Martínez JG, Mihoub J-B, Mills JA, Molina-Morales M, Moksnes A, Ozgul A, Parejo D, Pilard P, Poisbleau M, Rousset F, Rödel M-O, Scott D, Senar JC, Stefanescu C, Stokke BG, Kusano T, Tarka M, Tarwater CE, Thonicke K, Thorley J, Wilting A, Tryjanowski P, Merilä J, Sheldon BC, Møller P, Matthysen A, Janzen E, Dobson F, Visser FS, Beissinger ME, Courtiol SR, A., and, Kramer-Schadt. S. 2019. Adaptive responses of animals to climate change are most likely insufficient. *Nat Commun* 10, 1, 3109. 10.1038/s41467-019-10924-4 PMC6650445 31337752

[55] Reinelt L, Whitaker J, Kazakou E, Bonnal L, Bastianelli D, Bullock JM, Ostle NJ. Drought effects on root and shoot traits and their decomposability. Funct Ecol. 2023;37(4):1044–54. 10.1111/1365-2435.14261

[56] Ren G, Zhu C, Alam MS, Tokida T, Sakai H, Nakamura H, Usui Y, Zhu J, Hasegawa T, Jia Z. Response of soil, leaf endosphere and phyllosphere bacterial communities to elevated CO2 and soil temperature in a rice paddy. Plant Soil. 2015;392(1–2):27–44. 10.1007/s11104-015-2503-8

[57] Rolli E, Marasco R, Vigani G, Ettoumi B, Mapelli F, Deangelis ML, Gandolfi C, Casati E, Previtali F, Gerbino R, Pierotti Cei F, Borin S, Sorlini C, Zocchi G, Daffonchio D. Improved plant resistance to drought is promoted by the root-associated microbiome as a water stress-dependent trait. Environ Microbiol. 2015;17:2, 316–31. 24571749 10.1111/1462-2920.12439

[58] Sadok W, Lopez JR, Smith KP. Transpiration increases under high-temperature stress: potential mechanisms, trade-offs and prospects for crop resilience in a warming world. Plant Cell Environ. 2021;44:7, 2102–16. 33278035 10.1111/pce.13970

[59] Santos-Medellín C, Edwards J, Liechty Z, Nguyen B, Sundaresan V. 2017. Drought Stress Results in a Compartment-Specific Restructuring of the Rice Root-Associated Microbiomes. *mBio* 8, 4. 10.1128/mBio.00764-17 PMC5516253 28720730

[60] Schimel J, Balser TC, Wallenstein M. Microbial stress-response physiology and its implications for ecosystem function. Ecology. 2007;88(6):1386–94. 17601131 10.1890/06-0219

[61] Sheik CS, Beasley WH, Elshahed MS, Zhou X, Luo Y, Krumholz LR. Effect of warming and drought on grassland microbial communities. ISME J. 2011;5:10, 1692–700. 21451582 10.1038/ismej.2011.32 PMC3176507

[62] Staszel K, Lasota J, Błońska E. Effect of drought on root exudates from Quercus petraea and enzymatic activity of soil. Sci Rep. 2022;12:1. 35538167 10.1038/s41598-022-11754-z PMC9090927

[63] Su X, Su X, Zhou G, Du Z, Yang S, Ni M, Qin H, Huang Z, Zhou X, Deng J. Drought accelerated recalcitrant carbon loss by changing soil aggregation and microbial communities in a subtropical forest. Soil Biol Biochem. 2020;148:107898. 10.1016/j.soilbio.2020.107898

[64] Tiziani R, Miras-Moreno B, Malacrinò A, Vescio R, Lucini L, Mimmo T, Cesco S, Sorgonà A. Drought, heat, and their combination impact the root exudation patterns and rhizosphere microbiome in maize roots. Environ Exp Bot. 2022;203:105071. 10.1016/j.envexpbot.2022.105071

[65] Treseder KK, Lennon JT. Fungal traits that drive ecosystem dynamics on land. Microbiol Mol Biology Reviews: MMBR. 2015;79:2, 243–62. 25971588 10.1128/MMBR.00001-15 PMC4429240

[66] Vancov T, Keen B. Amplification of soil fungal community DNA using the ITS86F and ITS4 primers. FEMS Microbiol Lett. 2009;296(1):91–6. 19459948 10.1111/j.1574-6968.2009.01621.x

[67] Wagg C, Hautier Y, Pellkofer S, Banerjee S, Schmid B, van der Marcel GA. Heijden. 2021. Diversity and asynchrony in soil microbial communities stabilizes ecosystem functioning. *eLife Sciences Publications, Ltd* (Mar. 2021). 10.7554/eLife.62813 PMC7987343 33755017

[68] White TD, Bruns SB, Lee, Taylor JW, White TJ, Bruns TD, Lee SB. and J. W. Taylor. Amplification and direct sequencing of fungal ribosomal RNA genes for phylogenetics. PRC Protcols. Academic, 315–22.

[69] Wickham H. 2016. *Ggplot2*. *Elegant Graphics for Data Analysis*. Use R! Springer International Publishing; Imprint; Springer, Cham.

[70] Wipf HM-L, Bùi T-N, Coleman-Derr D. Distinguishing between the impacts of Heat and Drought stress on the Root Microbiome of Sorghum bicolor. Phytobiomes J. 2021;5:2, 166–76. 10.1094/PBIOMES-07-20-0052-R

[71] Xu L, Naylor D, Dong Z, Simmons T, Pierroz G, Hixson KK, Kim Y-M, Zink EM, Engbrecht KM, Wang Y, Gao C, DeGraaf S, Madera MA, Sievert JA, Hollingsworth J, Birdseye D, Scheller HV, Hutmacher R, Dahlberg J, Jansson C, Taylor JW, Lemaux PG, Coleman-Derr D. Drought delays development of the sorghum root microbiome and enriches for monoderm bacteria. Proc Natl Acad Sci USA. 2018;115:18, E4284–93. 29666229 10.1073/pnas.1717308115 PMC5939072

[72] Yang Cao. 2021. *microbiomeMarker*. Bioconductor.

[73] Zhang J, Nasir F, Kong Y, Tian L, Batool A, Bahadur A, Li X, Tian C. Drought stress shapes the root-associated bacterial and fungal community structure in soybean genotypes. Pak J Bot. 2017;49(5):1933–42.

[74] Zhang Y, Zhang Y, Xu W, Hu J, Zhang Z. Possible effects of temperature on bacterial communities in the rhizosphere of rice under different climatic regions. Arch Microbiol. 2022;204:4, 212. 35296917 10.1007/s00203-022-02812-1

[75] Zhou R, Yu X, Zhao T, Ottosen C-O, Rosenqvist E, Wu Z. Physiological analysis and transcriptome sequencing reveal the effects of combined cold and drought on tomato leaf. BMC Plant Biol. 2019;19:1. 31455231 10.1186/s12870-019-1982-9 PMC6712725

