# Supplementary material for: Oak seedling microbiome assembly under climate warming and drought
Source: Environ Microbiome. 2024 Aug 28;19:62. doi: 10.1186/s40793-024-00602-4 (PMC11360865; doi:10.1186/s40793-024-00602-4)
Supplement: Supplementary file 1 — Supplementary Material 1 [file 40793_2024_602_MOESM1_ESM.docx]

**Supplementary** **Information**


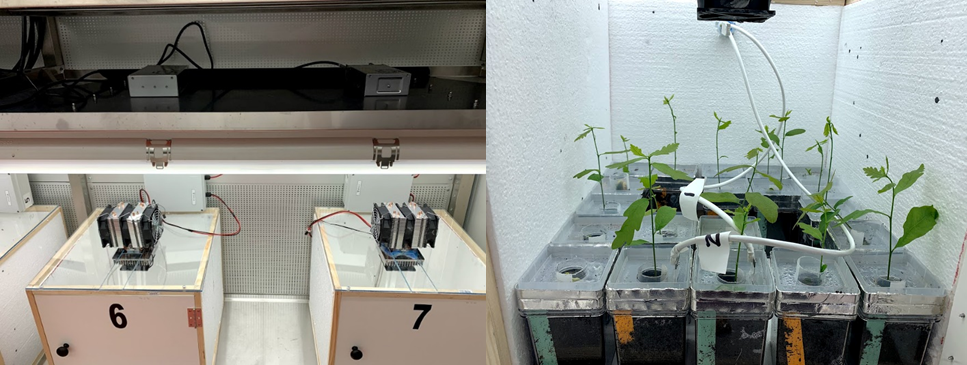


Figure S1: **Left**: Climate boxes with integrated humidity and temperature sensors. **Right**: Oak seedlings planted in soil with different soil moisture levels (green – low moisture, orange – high moisture) inside a climate box.

**The impact of temperature, soil moisture and their interaction on the fungal and bacterial community on rhizosphere and phyllosphere of oak.**

Table S1: The impact of temperature, soil moisture, and their interaction on the fungal and bacterial community on rhizosphere and phyllosphere of the oak Quercus robur using linear fixed effect models species richness, Shannon diversity and abundance and testing for significance with the ´Anova´ function (car package). R^2^-values were calculated with the function ´r.squaredGLMM´ (MuMln package) after running separate linear models for each fixed effect. The bacterial community composition on oak rhizosphere and phyllosphere as modelled using PERMANOVA (absolute count data; function ´adonis2´ from the package vegan). Significant values are indicated in bold.

|  |  | Soil Moisture | Temperature | Soil Moisture x  Temperature |
| --- | --- | --- | --- | --- |

| Rhizosphere |  | Measure | p | R^2^ | p | R^2^ | p | R^2^ |
| --- | --- | --- | --- | --- | --- | --- | --- | --- |
|  | Fungi | Species richness | 0.221 | 0.007 | 0.094 | 0.060 | 0.052 |  |
|  |  | Shannon diversity | 0.423 | 0.004 | **0.002** | 0.112 | 0.881 |  |
|  |  | Abundance | **0.030** | 0.102 | **0.019** | 0.119 | **0.020** |  |
|  |  | Community composition | **0.001** | 0.062 | **0.001** | 0.033 | **0.003** | 0.010 |
|  | Bacteria | Species richness | 0.078 | 0.017 | 0.623 | 0.002 | 0.712 |  |
|  |  | Shannon diversity | 0.692 | 0.001 | 0.989 | 0.001 | 0.895 |  |
|  |  | Abundance | **0.030** | 0.066 | **0.039** | 0.060 | 0.185 |  |
|  |  | Community composition | **0.001** | 0.149 | **0.001** | 0.022 | **0.048** | 0.009 |
| Phyllosphere | Fungi | Species richness | 0.498 | 0.004 | 0.550 | 0.013 | 0.115 |  |
|  |  | Shannon diversity | 0.138 | 0.024 | 0.520 | 0.008 | 0.238 |  |
|  |  | Abundance | **<0.001** | 0.305 | **0.004** | 0.066 | **<0.001** |  |
|  |  | Community composition | 0.269 | 0.012 | 0.221 | 0.068 | 0.260 | 0.015 |
|  | Bacteria | Species richness | 0.057 | 0.047 | 0.619 | 0.004 | 0.928 |  |
|  |  | Shannon diversity | **0.012** | 0.075 | 0.199 | 0.021 | 0.295 |  |
|  |  | Abundance | 0.199 | 0.023 | **0.001** | 0.153 | 0.214 |  |
|  |  | Community composition | **0.002** | 0.026 | **0.027** | 0.022 | 0.929 | 0.010 |

**Partitioning of the total β-diversity (Jaccard dissimilarity index) between the different treatments into species turnover and change in species numbers for the bacterial and fungal communities on oak phyllosphere and rhizosphere.**

*Table S2: Partitioning of the total beta diversity (βJAC, Jaccard dissimilarity index) between the different temperature treatments (15 °C – 20 °C, 20 °C – 25 °C, 15 °C – 25 °C) and between the different soil moisture treatments at the specific temperatures (15 °C high – low, 20 °C high - low, 25 °C high – low) into the components of species turnover (βJTU) and change in species numbers (βJNE) for the bacterial and fungal communities on both phyllosphere and rhizosphere.*

|  |  | **Phyllosphere** | | | **Rhizosphere** | | |
| --- | --- | --- | --- | --- | --- | --- | --- |
|  | **Comparison** | **βJAC** | **βJTU** | **βJNE** | **βJAC** | **βJTU** | **βJNE** |
| **Bacteria** | 15 °C – 20 °C | 0.9082 | 0.8892 | 0.0190 | 0.5014 | 0.4481 | 0.0533 |
|  | 20 °C – 25 °C | 0.8722 | 0.8378 | 0.0344 | 0.5111 | 0.4484 | 0.0627 |
|  | 15 °C – 25 °C | 0.8971 | 0.8366 | 0.0605 | 0.5034 | 0.4943 | 0.0091 |
|  | 15 °C: high - low | 0.9292 | 0.8300 | 0.0992 | 0.6264 | 0.5761 | 0.0503 |
|  | 20 °C: high - low | 0.9482 | 0.9207 | 0.0275 | 0.6555 | 0.6493 | 0.0062 |
|  | 25 °C: high - low | 0.8947 | 0.7349 | 0.1598 | 0.8610 | 0.5280 | 0.0333 |
| **Fungi** | 15 °C – 20 °C | 0.8856 | 0.8169 | 0.0687 | 0.7292 | 0.6787 | 0.0524 |
|  | 20 °C – 25 °C | 0.8511 | 0.8387 | 0.0124 | 0.7112 | 0.6814 | 0.0298 |
|  | 15 °C – 25 °C | 0.8975 | 0.8526 | 0.0449 | 0.7319 | 0.7117 | 0.0202 |
|  | 15 °C: high - low | 0.8661 | 0.8468 | 0.0193 | 0.7376 | 0.6746 | 0.0630 |
|  | 20 °C: high - low | 0.7381 | 0.4590 | 0.2791 | 0.7335 | 0.7278 | 0.0057 |
|  | 25 °C: high - low | 0.8125 | 0.7731 | 0.0394 | 0.7324 | 0.7117 | 0.0207 |
